# Disruptions in Liver Function among Cancer Patients and Patients Treated with Tyrosine Kinase Inhibiting Drugs: Comparisons of Two Population-Based Databases

**DOI:** 10.1155/2013/358285

**Published:** 2013-04-21

**Authors:** Sarah H. Landis, Beth L. Nordstrom, Leah B. Sansbury, Sumitra Shantakumar, Samantha A. St. Laurent, Kathy H. Fraeman, Jeanenne J. Nelson

**Affiliations:** ^1^Worldwide Epidemiology, GlaxoSmithKline, Stockley Park West, Middlesex, London UB11 1BU, UK; ^2^Epidemiology and Database Analytics, United BioSource Corporation, Lexington, MA 02420, USA; ^3^Worldwide Epidemiology, GlaxoSmithKline, Research Triangle Park, NC 27709-3398, USA; ^4^Epidemiology and Database Analytics, United BioSource Corporation, Bethesda, MD 20814, USA

## Abstract

Liver toxicity is a recognized adverse event associated with small molecule tyrosine kinase inhibitors (TKIs). Electronic Medical Record (EMR) databases offer the most precise data to investigate the rate of liver function test (LFT) elevations; however, they can be limited in sample size and costly to access and analyze. Health insurance claims databases often contain larger samples sizes but may lack key health information. We evaluated the feasibility of utilizing a large claims database to calculate incidence rates (IRs) of LFT elevations among a general cohort of cancer patients and a cohort of patients treated with TKIs by comparing the results to a “gold standard” oncology-specific EMR database. IRs for the TKI cohorts were very similar between the two databases; however, IRs were higher in the EMR database for the cancer cohorts. Possible explanations for these differences include lack of specificity when defining a cancer case, poor capture of laboratory data, or inaccurate assessment of person-time in the insurance claims database. This study suggests that insurance claims data may provide reliable results when investigating liver toxicities associated with oncology drug exposure; however, there are limitations when assessing laboratory outcomes for cohorts defined solely by disease status.

## 1. Introduction

Therapeutic agents that target cancer-specific molecules and signalling pathways have become increasingly integrated into cancer care in recent years. Activation of tyrosine kinases plays a critical role in modulation of growth factor signalling, such as increased cell proliferation and growth, induced antiapoptotic effects, and promotion of angiogenesis and metastasis, and as a result, these protein kinases are key targets for inhibition [1, 2]. Tyrosine kinases can be further classified as receptor kinases and nonreceptor protein kinases [2]. Small-molecule inhibitors of tyrosine kinase target a number of receptors, including BCR-ABLE, c-KIT, PDGFR, EGFR, and FLT-3 [2]. Currently, there are six small molecule TKIs approved by the FDA: imatinib (Gleevec, Glivec), gefitinib (Iressa), erlotinib (Tarceva), dasatinib (Sprycel), lapatinib (Tykerb, Tyverb), and nilotinib (Tasigna).

The liver plays a major part in metabolic and excretory functions, including a key role in the metabolism of a number of anticancer cytotoxics and biologic agents, causing drug inactivation or activation of a prodrug. In turn, chemotherapy and biological agents can induce liver injury or dysfunction, which can manifest in abnormal serum liver biochemistry [3]. Drug-related adverse events of severe liver toxicity are defined by Grade 3 (>5.0–20.0× ULN) or Grade 4 (≥20× ULN) elevations in alanine transaminase (ALT) or aspartate transaminase (AST), or Grade 3 (>3.0–10.0× ULN) or Grade 4 (>10.0× ULN) elevations for total bilirubin (BILI) [4]. Severe liver toxicity, known as hepatotoxicity, is a recognized adverse event for the small molecule TKIs, although its incidence is relatively rare in most TKI clinical trials [5–9]. However, some clinical trials have reported a much higher incidence of hepatotoxicity, including up to 14% of gefitinib users and 6% of imatinib users [10, 11].

In order to benchmark the incidence of liver enzyme elevations observed in TKI clinical trials against expected rates, it is crucial to understand the background incidence rates of elevations in the general cancer patient population and in patients receiving TKIs. Population-based data sources commonly used to evaluate incidence rates include electronic medical record (EMR) databases and health insurance claims databases. Electronic medical records capture a complete patient record of diagnoses, treatments, hospitalizations, laboratory, and pathology results and thus serve as rich dataset for conducting research. EMRs, however, can be limited in the number of captured patients which may limit their usefulness for analyzing rare diseases and rare outcomes. In contrast, insurance claims databases often contain millions of patients, but the data captured can be limited since they are collected primarily for administrative and reimbursement purposes and not to create a clinical record. Furthermore, insurance claims databases may not capture detailed information regarding diagnoses (e.g., cancer stage and histology), and although laboratory tests are covered, test results are not always available in the claims database. In this study, we evaluated the feasibility of characterizing liver enzyme elevations in cancer patients being treated in real-world, population-based settings by exploring two databases: an insurance claims database and an oncology-specific EMR database.

The aims of this study were to (1) describe LFT availability in an insurance claims database and an EMR database; (2) calculate the incidence rate (IR) of LFT elevations in the insurance claims database and the EMR database; (3) compare the results obtained from the insurance claims database to the “gold standard” EMR database to describe the utility of using insurance claims databases to estimate incidence rates of laboratory abnormalities in an oncology setting. In order to address these aims, we utilized two patient cohorts: a general cohort of patients with selected solid tumors and a small molecule TKI drug treated cohort of patients with any cancer type. Although this study focused on these specific populations, we believe that the findings may be generally applicable to other pharmacoepidemiology studies utilizing laboratory-based claims data to investigate other laboratory events and classes of marketed oncology drugs.

## 2. Methods

### 2.1. Database Descriptions

#### 2.1.1. EMR Database

The Varian Medical Oncology database of outpatient oncology practices was considered the “gold standard” EMR database for this study. At the time the study was conducted (data extraction September 2008), this oncology-specific EMR system contained data on more than 185,000 cancer patients from 18 participating oncology practices in 15 states across the United States. During each patient visit, clinic staff enter data about the visit, including diagnoses, treatments, and other relevant information. Diagnoses are entered as ICD-9-CM codes, supplemented with International Classification of Diseases for Oncology (ICD-O) codes and staging information for a subset of patients. Treatment data include orders or prescriptions for medications, with specifics such as dose and route, as well as duration of supply of oral medications, and amount and timing of drugs administered in the clinic. Laboratory results are typically fed directly from the lab into the EMR system; data include the date of the test, lab test name, result, units, and normal range. The data used for the present study were deidentified, as required by the Health Insurance Portability and Accountability Act (HIPAA).

#### 2.1.2. Insurance Claims Database

We used the Clinformatics for Data Mart, a product of OptumInsight Life Sciences, as the insurance claims database for this study. This database is a comprehensive, deidentified US healthcare claims database that contains the aggregated health claims experience of over 24 million individuals covered by the health insurance program. It contains only those individuals covered for which there exists a combined benefit structure including medical and prescription coverage. Overall, it is representative of the nonelderly, insurance-carrying population in the United States, but it also contains information on several hundred thousand individuals enrolled in the government-sponsored Medicare Advantage program, a health and medical services program for persons 65 years and older, and the Managed Medicaid program, a health and medical services program for certain individuals and families with low incomes and few resources. The insurance claims database is geographically diverse, including data for members in all 50 states. It contains inpatient, outpatient, and pharmacy claims and integrates the outpatient test result values for lab tests processed by the two largest US national lab vendors. The data used for the present study were deidentified, as required by the Health Insurance Portability and Accountability Act (HIPAA).

### 2.2. Study Populations

**Figure 1:**
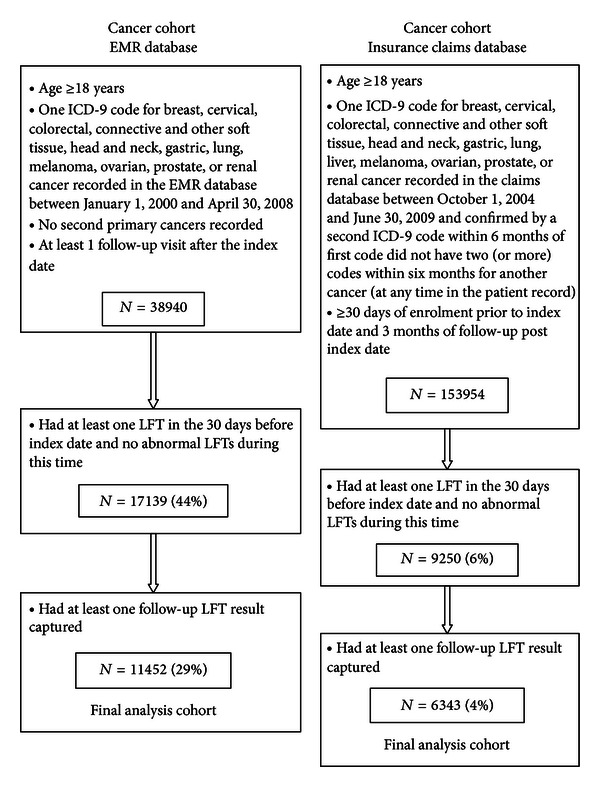
Cohort selection, cancer cohorts.

A summary of the cancer cohort study populations can be found in Figure 1. As the EMR study was considered the “gold standard” for analyzing LFT elevations, we constructed the insurance claims study cohorts to mimic the cohorts created in the EMR study as best possible. Briefly, the cancer cohorts were defined by having a qualifying cancer diagnosis for breast, cervical, colorectal, connective and other soft tissue, head and neck, gastric, liver (insurance claims cohort only), lung, melanoma, ovarian, prostate, or renal cancer determined from ICD-9-CM codes. As the EMR database included patients actively undergoing cancer treatment, only one ICD-9-CM cancer code was required to define the cancer type, while patients needed two ICD-9-CM codes for the same cancer within a 6-month period to be eligible for the insurance claims cohort. The EMR database excluded patients with a recorded second primary cancer. In order to implement this exclusion in the insurance claims database, patients with one or more additional pairs of ICD-9-CM codes within a 6-month period (beyond the qualifying cancer diagnosis) were excluded from the cohort and analyses.

**Figure 2:**
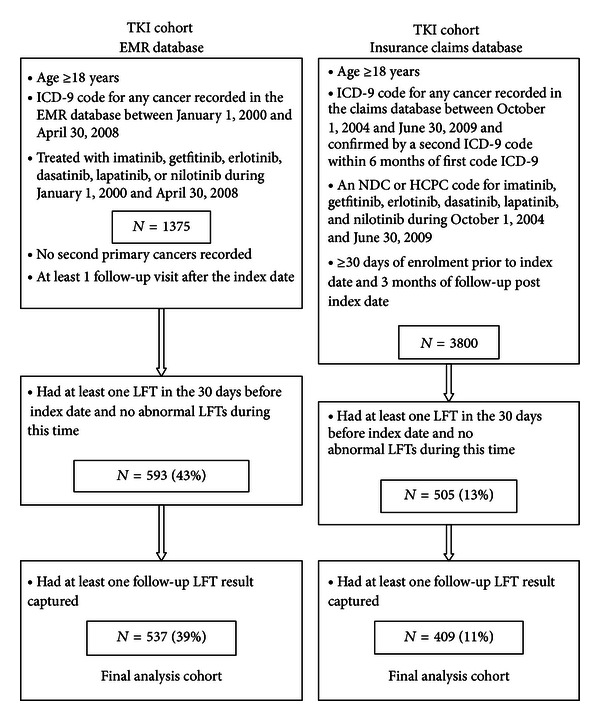
Cohort selection, TKI cohorts.

Figure 2 describes the TKI cohort study populations. The TKI cohort included patients with *any cancer* who were treated with one or more TKI agents including imatinib, gefitinib, erlotinib, dasatinib, lapatinib, and nilotinib. A small proportion of patients that had used two TKIs during the study period (4%) were classified according to the first TKI used. Patients in the EMR database with multiple primary cancer diagnoses were excluded as done in the EMR cancer cohort. However, the multiple primary exclusion was not similarly applied to the insurance claims TKI cohort, as a large proportion of eligible patients were lost when this was applied.

Patients were followed from the index date (date of a first qualifying cancer diagnosis or date of first prescription of TKI, depending on the cohort) through the last visit in the database due to either death, drop out of the health plan, or through the end of the analysis period (April 30, 2008 for the EMR database and June 30, 2009 for the insurance claims database), whichever came first. Several additional eligibility criteria regarding availability of LFTs in the database and length of database enrollment around the index date were applied universally across both the cancer and TKI cohorts in both databases. In order to ensure that patients had laboratory data captured in their record and to exclude any patients with prevalent LFT elevations (prior to baseline), all included patients had to have (1) at least one LFT measured ≤30 days before index date and no abnormal elevations during that time and (2) at least one follow-up LFT result at any point in the record after the index date. Follow-up time criteria of at least one follow-up visit recorded (EMR databases) or at least one month of enrolment prior to and three months post index date (insurance claims database) were instituted to ensure that patients were continuously enrolled in their respective database during the analyses time period and to limit missing LFTs due to switching of insurance companies.

Patients who qualified for more than one of the cohorts (cancer and TKI) were allowed to contribute to each, often with different index dates for each cohort. Demographics for ineligible patients were captured in order to make comparisons with the eligible populations.

### 2.3. Outcome Definitions

The outcome of interest is the incidence of elevated LFTs which was defined identically in all cohorts. Incidence rates (IRs) and 95% confidence intervals (CIs) were calculated for elevations of ALT, AST, ALP, and total bilirubin and were defined as a value measured after the index date that was above selected cut points of the upper limit of normal (ULN) for the test. Combinations of LFTs according to probable Hy's Law (ALT or AST ≥ 3× ULN, ALP < 2× ULN, and bilirubin ≥ 2× ULN) which denote possible clinically significant liver abnormalities were also examined [12]. For the combination, each component LFT was required to occur on the same day.

For the cancer cohorts, patients were followed for elevations in LFTs from the index date (date of first qualifying cancer diagnosis) through the last LFT measurement before the database cutoff. For the TKI cohorts, LFT elevations were first calculated considering all follow-up time from initiation of TKI through the last LFT measurement before the database cutoff regardless of whether patients were actively taking the drug (i.e., all patient time after the index date was considered to be “drug-exposed” time). Secondly, LFT elevations were calculated during distinct periods of drug exposure (and nonexposure) during followup. The duration of exposure to the oral TKI agents was determined by a combination of available variables for days supply, dispensed quantity, administration frequency (quantity/time), and number of refills. For example, an oral drug with a 30-day supply and 2 refills had a total duration of 90 days. We used a 45-day or more gap in refill or administration of treatment to define time “off” of treatment. We calculated *χ* ^2^ statistics to determine whether significant differences in categorical variables were present between eligible and ineligible patients within each cohort from a single database. However, the comparisons between the insurance claims and EMR databases are only descriptive with no formal testing. All analyses were conducted using SAS version 9.1.

## 3. Results

### 3.1. Patient Characteristics

#### 3.1.1. EMR Cancer Cohort

Among the 38,940 patients who had an ICD-9-CM code for one of the solid tumors of interest, 11,452 (29%) met the additional LFT and follow-up time criteria (Figure 1). Among these eligible patients, two-thirds were females and the mean age at diagnosis was 62 years (standard deviation (SD) = 13) (Table 1). Breast (34.7%), lung (27.5%), and colorectal (17.9%) cancers were the most common cancer types observed.

The most notable difference between eligible and ineligible patients in the EMR cancer cohort was the distribution in the type of health insurance. There was a lower proportion of eligible patients with private health insurance (14.9% versus 24.1%) and a greater proportion with other/unknown health insurance (53.1% versus 37.1%). The distribution of cancer type among the eligible and ineligible patients was fairly similar with breast, lung, and colorectal being the most common cancers among both groups.

#### 3.1.2. EMR TKI Cohort

Of the 1,375 patients who had received one of the TKIs of interest, 537(39%) were eligible for the analyses (Figure 2). Among the eligible patients, the median age was 63 years (SD = 14), and 53% were females (Table 1). The most common small molecule TKI used was erlotinib (55.1%), followed by imatinib (31.3%). The eligible and ineligible patients were similar with regard to distribution of gender, age, health insurance type, and TKI prescribed.

#### 3.1.3. Insurance Claims Cancer Cohort

Among the 153,954 patients who met the case definition for cancer (two or more ICD-9-CM codes for the same cancer within six months), 6,343 (4%) met the additional LFT and follow-up criteria (Figure 1). Sixty-four percent of the eligible patients were females (63.7%) and the mean age was 57 years (SD = 10) (Table 2). The eligible group was more likely to be females than the ineligible group and slight differences in the most common cancers were noted between the two populations.

#### 3.1.4. Insurance Claims TKI Cohort

Among the 3,800 patients who met the enrolment criteria for the insurance claims database TKI cohort, 409 (11%) patients were eligible for the LFT analysis (Figure 2). As observed in the cancer cohort, the majority of eligible patients in the TKI cohort were female (57.2%); however, the difference was not as extreme (Table 2). Erlotinib (57.7%) and imatinib (26.9%) were the most common small molecule TKIs received among the patients in the TKI cohort.

The most notable differences observed between the eligible and ineligible groups in the insurance claims TKI cohort were insurance type (eligible population had lower proportion of IND and PPO coverage and higher EPO and POS coverage). This insurance type difference likely reflects the fact that certain insurers use commercial laboratory networks that automatically provide results back to the insurer, thus increasing LFT availability for patients with those insurance types.

#### 3.1.5. Comparison of Eligible Patient Characteristics in the Cancer Cohorts

The insurance claims database was able to replicate the EMR eligible population with regard to gender distribution; however, eligible patients in the insurance claims database were slightly younger (mean 57 years versus 62 years). This age difference is probably because the insurance claims database represents primarily nonelderly, commercially insured adults whereas the EMR system may represent a broader patient population with both commercial insurance and Medicare coverage. While the most common cancers among the eligible population in the EMR database were breast (34.7%), lung (27.5%), and colorectal (17.9%), the insurance claims database commonest cancers were breast (48%), prostate (18%), and colorectal (12%). The higher prevalence of prostate cancer observed in the insurance claims database is likely an artifact of picking up ICD-9-CM codes for prostate cancer screening (PSA testing) rather than actual confirmed cases of prostate cancer.

#### 3.1.6. Comparison of Eligible Patient Characteristics in the TKI Cohorts

The insurance claims TKI eligible cohort was slightly more likely to be females (57%) compared to the EMR TKI eligible cohort (53%) and, as observed with the cancer cohort, was slightly younger (54 years versus 63 years). In addition, the insurance claims TKI cohort had fewer patients exposed to gefitinib (3.4% versus 11.2%, resp.), while slightly more patients were exposed to lapatinib (11.7% versus 0.4%), respectively, when compared to the TKI cohort in the EMR database.

#### 3.1.7. Comparison of IRs in the Cancer Cohorts

A comparison of IRs for selected LFT elevation thresholds for the two cancer cohorts is provided in Table 3. The cumulative incidence (CI) and incidence rates (IRs) for most LFT elevations were several magnitudes higher, as much as ten times higher, in the EMR database compared to the insurance claims database. For example, the IRs in the EMR database for ALT and AST > 3× ULN was 3.8 per 100 person-years (1.0–10.0) and 3.1 per 100 person-years (0.6–8.9), respectively, compared to 0.4 per 100 person-years (0.3–0.6) and 0.3 per 100 person-years (0.2–0.4), respectively, in the insurance claims database (Table 3). Similar differences were observed for ALP > 2× ULN and serum bilirubin > 1.5× ULN. The differences were even greater at upper elevations or the combination endpoint; however, these were based on very small incidence rates and the absolute differences were not as clinically significant.

#### 3.1.8. Comparison of IRs in the TKI Cohorts

Unlike the comparison of the cancer cohorts, the insurance claims TKI cohort had IRs of similar magnitude as those observed in the EMR TKI cohort (Table 4). The EMR database provided slightly higher incidence rates for patients with >1.5× ULN for BILI, but the remainder of the thresholds investigated and the combination endpoint were nearly identical. 

In the analysis that stratified IRs by time “on” and “off” TKI, we observed very similar IRs for LFT elevations among patients currently “on” TKI drugs (data not shown). However, the sample size for these analyses was limited which decreases the ability to make firm conclusions with regard to these data.

## 4. Discussion and Conclusions

This study assessed the feasibility of using an insurance claims database to examine LFT elevations in cancer patients and patients receiving TKIs by comparing the patient population and IRs of LFT elevations to those obtained in a “gold standard” oncology-specific EMR database.

### 4.1. Database Comparison in terms Capture of Liver Function Tests

After applying several inclusions criteria regarding availability of LFTs, only 5% and 13% of the insurance claims database cancer and TKI cohorts, respectively, were eligible to be included in the analysis. For the EMR database, the proportion of eligible patients was greater in both cohorts (29% and 39% for the cancer and TKI cohorts, resp.). It is important to recognize that these percentages do not reflect actual screening rates in medical practice (or more frequent screening in the clinics that participate in the EMR database), but rather how well laboratory results are captured within the respective databases. In addition, we required an LFT to be captured within a narrow window of only 30 days prior to the index date in order to exclude patients with prevalent LFT elevations immediately prior to cohort entry. A sensitivity analysis in the insurance claims database explored wider ranges of baseline time before and after the index date (data not shown). As expected, these wider time windows resulted in greater eligible sample sizes; however, the patient characteristics and LFT results were similar regardless of the baseline window utilised, and thus we utilised the 30 days prior window as this is in line with the design of the gold standard EMR cohorts. These eligibility data suggest, as expected, that the EMR cohort has better capture of laboratory test results. In addition, the higher availability of LFTs among the TKI cohorts regardless of database may reflect more frequent screening of patients who are prescribed these drugs, given the known class association with hepatotoxicity.

### 4.2. Database Comparison in terms of Liver Function Test Incidence Rates

The insurance claims cancer cohort produced IRs that were considerably lower than the EMR cancer cohort, whereas IRs for the TKI cohorts were generally comparable across databases. There are several potential explanations for why IR differences across the two databases might be observed in the cancer cohort but not the TKI cohort.

As discussed earlier, we believe that the EMR database has more complete capture of LFTs, possibly resulting in greater numbers of incident LFT elevations being contributed to the IR numerator. However, it is unlikely that this would explain the observed IR disparities in only the cancer cohort, since the improved LFT capture was seen in both the cancer and the TKI cohorts.

A second possibility is that the insurance claims eligible population differed from the eligible EMR population with regard to key characteristics that may influence the likelihood of having an LFT elevation. Indeed, some small differences in age and cancer site distribution were observed between the cancer cohort eligible populations; however, similar age differences were also noted in the TKI cohorts, and primary cancer site is not a strong predictor of LFT elevations, unless possibly through a pathway of enhance proclivity to metastasize to the liver. We were not able to investigate the influence of liver metastases as this variable was not available in the insurance claims database; however, in the EMR databases, this represented a very small proportion of the overall cancer cohort or TKI cohort populations.

A third potential explanation is that the insurance claims database captures all patient follow-up time until a person has a change in health plan, whereas the EMR database reflects only the active cancer treatment period. Thus, the insurance claims database likely includes an inflated person-time denominator that includes “noncancer” time after patients have gone into remission/cure and are not likely to have elevated LFTs. This phenomenon may have less influence on the TKI cohort, where the index date of TKI initiation represents a later stage in the cancer continuum as these agents are mostly used in the metastatic setting when patients are closer to death/end of insurance plan enrolment.

The most probable explanation relates to how the cohorts were defined within the insurance claims database. The EMR cancer cohort captured only patients actively undergoing cancer treatment, and thus we are certain that these patients have cancer. However, cancer cases in the insurance claims cancer cohort were identified using a case definition of two ICD-9-CM codes for the same cancer within six months. It is possible that this definition was not highly specific and resulted in overinflation of the IR denominator by including person-time of “false” cancer cases or people undergoing a screening or diagnostic workup for suspected cancer that was not deemed to be malignant. In an analysis examining the accuracy of cancer case identification in a claims database, Setoguchi and colleagues compared several increasingly detailed cancer case-definitions against confirmed cancer diagnoses from cancer registry data in the United States [13]. They observed positive predictive values (PPVs) between 18.82% and 81.74% [13], illustrating the relatively low sensitivity that can occur when attempting to identify cancer cases in claims databases. Further, in an attempt to mimic the EMR cancer cohort's exclusion of patients with a second primary cancer, we also excluded patients in the insurance claims cancer cohort who met the criteria of two ICD-9-CM codes for another cancer during the same time period as the index cancer. As primary cancers cannot always be easily distinguished from metastases in an insurance claims database, this rule may have actually resulted in exclusion of patients most likely to be true cancer cases with a second set of codes indicative of a metastatic event. Indeed, when examining the baseline characteristics of the insurance claim TKI cohort (Table 2), it appeared that 45% of patients met the criteria for two cancers, 22% for three cancers, and 7.5% for four or more cancers.

However, these concerns are less of an issue in the insurance claims TKI cohort, as inclusion in that cohort required a prescription for a TKI, which are only used in oncology treatment. Furthermore, since we selected the date of the first TKI prescription as the index date, we have a higher confidence that these are true cancer patients who are actively in treatment (at least for the immediate time period after index date). Taken together, these arguments suggest that when patients are defined in an insurance claims database based upon receiving a particular anticancer treatment in addition to cancer codes, the claims database is more accurate compared to identifying cancer patients using only ICD-9-CM codes for diagnosis.

### 4.3. Limitations of the Analysis

A general limitation of using a claims database to study cancer patients is the lack of data on key cancer related variables that may be important factors in choice of treatment, cancer prognosis, or risk of adverse events. For example, the insurance claims database does not include information on the presence of liver metastasis, which may be contributing factor for liver function elevations. This data was available in the EMR cohorts and represented a very small proportion of either cohort; thus, the lack of this variable in insurance claims databases should perhaps not preclude the use of these databases for studying LFTs.

Both databases primarily capture labs ordered on an outpatient basis and therefore may underestimate elevations recorded during inpatient hospital stays. Both databases utilized requirements of follow-up time (at least one further visit in the EMR database or at least three months of follow-up enrollment in the insurance claims database) which may result in immortal person-time. Since all patients in the cohorts have similar requirements, the only limitation this creates is that the included patients might not be representative of all cancer and TKI patients, since those who disenroll early or those who die shortly after initiating treatment may have had different LFT patterns than those seen in these cohorts. Finally, since all data are deidentified, we have no way of knowing if an individual who was treated at an oncology clinic in the Varian system also had an insurance plan captured in the insurance claims database and thus may have been included twice. We do not believe that there are a large number of individuals who would be included in both.

The outcome of elevated liver function is considered to be an adverse event when the elevation can be directly attributed to the use of a particular medication. While neither the EMR nor insurance claims databases can be used to definitively associate a recorded elevation with a particular drug, we were able to measure LFT elevations by time “on” and “off” TKI in both cohorts (data not shown). The sample size for analysis in both of the cohorts was limited, thus decreasing the ability to draw conclusions from the analysis. However, the IRs for TKI elevations observed in the EMR database were similar in magnitude to those observed in the insurance claims database (data not shown).

## 5. Conclusion

This study highlights the strengths and limitations of using an insurance claims databases to estimate the incidence rates of a laboratory abnormality in cancer patients in general, and those being treated with a particular class of antineoplastic drugs. The ability to compare the results derived from the insurance claims database to a “gold standard” oncology database is a key attribute of this study and illuminates some limitations to claims databases which may bias incidence results. These limitations include difficulty in accurately identifying “true” cancer patients in a claims database as well as possible underreporting of laboratory results. Interestingly, the results for the insurance claims database and EMR database were highly comparable for the TKI cohort, replicating other studies demonstrating that case definitions which incorporate both disease and treatment criteria are more accurate at identifying cancer patients under active treatment. While this study looked at only one claims database and one set of laboratory tests, we believe that the results may be widely applicable to other claims databases and other laboratory-defined events in the oncology setting.

## Figures and Tables

**Table 1:** Baseline demographics for ineligible patients and eligible patients: EMR database.

	Cancer cohort					TKI cohort				
	Ineligible patients*		Eligible patients**			Ineligible patients*		Eligible patients**		
	(*N* = 27,488)		(*N* = 11,452)		*P* value	(*N* = 838)		(*N* = 537)		*P* value
	*N*	%	*N*	%		*N*	%	*N*	%	
Gender										
Male	9,855	35.9	3,885	33.9	0.0003	418	49.9	254	47.3	0.35
Female	17,633	64.1	7,567	66.1		420	50.1	283	52.7	
Age at diagnosis or start of TKI, years (mean (SD))										
	62.2 (13.28)		62.4 (13.21)		0.46	62.2 (14.04)		62.6 (14.30)		0.67
Health insurance type										
Private	6,613	24.1	1,706	14.9	<0.0001	174	20.8	108	20.1	0.26
Public	3,404	12.4	1,301	11.4		115	13.7	81	15.1	
Self	177	0.6	19	0.2		6	0.7	3	0.6	
Mixed	7,089	25.8	2,345	20.5		235	28.0	175	32.6	
Other/unknown	10,205	37.1	6,081	53.1		308	36.8	170	31.7	
Cancer type										
Breast	9,964	36.2	3,973	34.7	<0.0001	—	—	—	—	
Cervical	474	1.7	130	1.1		—	—	—	—	
Colorectal	4,019	14.6	2,054	17.9		—	—	—	—	
Connective and other soft tissue	573	2.1	159	1.4		—	—	—	—	
Head and neck	872	3.2	352	3.1		—	—	—	—	
Gastric	407	1.5	165	1.4		—	—	—	—	
Lung	6,306	22.9	3,152	27.5		—	—	—	—	
Melanoma	950	3.5	260	2.3		—	—	—	—	
Ovarian	980	3.6	460	4.0		—	—	—	—	
Prostate	2,308	8.4	504	4.4		—	—	—	—	
Renal	635	2.3	243	2.1		—	—	—	—	
TKI type										
Dasatinib	—	—	—	—		19	2.3	9	1.7	0.088
Erlotinib	—	—	—	—		435	51.9	296	55.1	
Gefitinib	—	—	—	—		76	9.1	60	11.2	
Imatinib	—	—	—	—		289	34.5	168	31.3	
Lapatinib	—	—	—	—		16	1.9	2	0.4	
Nilotinib	—	—	—	—		3	0.4	2	0.4	
Follow-up time (months); mean (SD)			21 (19)					11 (12)		

*Ineligible patients did not meet the eligibility requirements for the cohort (see Figures 1 and  2). **Eligible patients had to have a normal baseline LFT value taken ≤30 days of index date and one follow-up LFT after index date. SD: standard deviation; TKI: tyrosine kinase inhibitor.

**Table 2:** Baseline demographics for ineligible patients and eligible patients: insurance claims database.

	Cancer cohort					TKI cohort				
	Ineligible patients*		Eligible patients**			Ineligible patients*		Eligible patients**		
	(*N* = 113,408)		(*N* = 6,343)		*P* value	(*N* = 2,782)		(*N* = 409)		*P* value
	*N*	%	*N*	%		*N*	%	*N*	%	
Gender										
Male	54,507	48.1	2301	36.3		1,277	45.9	175	42.8	
Female	58,874	51.9	4041	63.7	<0.0001	1,503	54.0	234	57.2	0.2326
Age at first cancer or age at start of TKI (years); mean (SD)										
	—		57 (10)			—		54 (10)		
Insurance type										
EPO	8,799	7.8	844	13.3	<0.0001	254	9.1	59	14.4	<0.0001
HMO	19,154	16.9	1,300	20.5		332	11.9	56	13.7	
IND	11,206	9.9	58	0.9		304	10.9	2	0.5	
OTH	1,179	1.0	1	0		2	0.1	0	0	
POS	55,683	49.1	3,551	56.0		1,471	52.9	250	61.1	
PPO	17,387	15.3	589	9.3		419	15.1	42	10.3	
Cancer type										
Breast	37,479	33.0	3,026	47.7	<0.0001	—		—		
Cervical	1,567	1.4	49	0.8		—		—		
Colorectal	10,305	9.1	773	12.2		—		—		
Connective and other soft tissue	1,272	1.1	37	0.6		—		—		
Gastric	503	0.4	33	0.5		—		—		
Head/neck	3,575	3.2	108	1.7		—		—		
Liver	751	0.7	81	1.3		—		—		
Lung	5,646	5.0	243	3.8		—		—		
Melanoma	12,962	11.4	466	7.3		—		—		
Ovarian	2,009	1.8	126	2.0		—		—		
Prostate	33,100	29.2	1,145	18.1		—		—		
Renal	4,239	3.7	256	4.0		—		—		
No. of unique cancers^†^										
One	—		—			709	25.5	92	22.5	0.3656
Two	—		—			1,261	45.3	181	44.3	
Three	—		—			601	21.6	101	24.7	
Four	—		—			165	5.9	24	5.9	
Five	—		—			29	1.0	6	1.5	
≥Six	—		—			17	0.6	5	1.2	
TKI type										
Dasatinib	—		—			26	0.9	1	0.2	0.4876
Erlotinib	—		—			1647	59.2	236	57.7	
Gefitinib	—		—			88	3.2	14	3.4	
Imatinib	—		—			734	26.4	110	26.9	
Lapatinib	—		—			279	10.0	18	11.7	
Nilotinib	—		—			8	0.3	—	—	
Follow-up time (months); mean (SD)	—		25 (16)			—		14 (12)		

*Ineligible patients did not meet the eligibility requirements for the cohort (see Figures 1 and  2). **Eligible patients had to have a normal baseline LFT value taken ≤30 days of index date and one follow-up LFT after index date. ^†^We are unable to distinguish diagnoses of multiple primaries from metastases in the claims data. EPO: exclusive provider organization; HMO: Health Maintenance Organization; IND: indemnity; OTH: other; POS: point of service; PPO: preferred provider organization; SD: standard deviation; TKI: tyrosine kinase inhibitor.

**Table 3:** Incidence of first occurrence of each degree of elevation for multiple liver function tests, cancer cohorts.

Liver function test	EMR database					Insurance claims database				
		Patients with elevation			Incidence rate per		Patients with elevation			
	Patients evaluated*	*N*	%	PY	100 PY (95% CI)	Patients evaluated*	*N*	%	PY	Incidence rate per 100 PY (95% CI)
ALT or SGPT (U/L)										
>3× ULN	11419	688	6.0	17987	3.8 (1.0–10.0)	5771	36	0.6	8190	0.4 (0.3–0.6)
>5× ULN		278	2.4	18357	1.5 (0.1–6.4)		8	0.1	8216	0.1 (0–0.2)
>10× ULN		95	0.8	18532	0.5 (0–4.7)		3	0.1	8220	0.04 (0–0.1)
>20× ULN		30	0.3	18599	0.2 (0–4.0)		0	0	8220	—
AST or SGOT (U/L)										
>3× ULN	11425	557	4.9	18212	3.1 (0.6–8.9)	5876	27	0.5	8377	0.3 (0.2–0.4)
>5× ULN		254	2.2	18450	1.4 (0.1–6.2)		12	0.2	8383	0.1 (0.1–0.2)
>10× ULN		99	0.9	18561	0.5 (0–4.7)		4	0.1	8390	0.05 (0–0.1)
>20× ULN		47	0.4	18597	0.3 (0–4.2)		1	0.02	8394	0.01 (0–0.1)
ALP (U/L)										
>2× ULN	11384	583	5.1	18354	3.2 (0.7–9.0)	5922	32	0.5	8403	0.4 (0.2–0.5)
>3× ULN		301	2.6	18515	1.6 (0.1–6.6)		14	0.2	8417	0.2 (0.1–0.3)
>5× ULN		124	1.1	18584	0.7 (0–5.0)		4	0.1	8421	0.05 (0–0.1)
Serum bilirubin, total (mg/dL)										
>1.5× ULN	11439	548	4.8	18222	3.0 (0.6–8.8)	5953	33	0.6	8471	0.4 (0.3–0.5)
>2× ULN		309	2.7	18444	1.7 (0.2–6.7)		14	0.2	8483	0.2 (0.1–0.3)
>5× ULN		98	0.9	18597	0.5 (0–4.7)		4	0.1	8489	0.05 (0–0.1)
>10× ULN		47	0.4	18614	0.3 (0–4.2)		1	0.02	8491	0.01 (0–0.1)
LFT combinations of Hy's Law^†^										
	11372	52	0.5	18559	0.3 (0–4.3)	5526	2	0.04	7905	0.03 (0–0.1)

*N evaluated: number of eligible patients with at least one follow-up measure of the corresponding LFT. ^†^Hy's Law = ALT or AST > 3× ULN, ALP < 2× ULN, and Bilirubin > 2× ULN. ULN: upper limit of normal; ALP: alkaline phosphatase; ALT: alanine aminotransferase; PY: person-years; SGPT: serum glutamic pyruvic transaminase; SGOT: serum glutamate-oxaloacetate transaminase.

**Table 4:** Incidence of first occurrence of each degree of elevation for multiple liver function tests, TKI cohorts.

Liver function tests (LFTs)	EMR database					Insurance claims database				
		Patients with elevation					Patients with elevation			
	Patients evaluated (*N*)*	N**	%^†^	PY	Incidence rate per 100 PY (95% CI)	Patients evaluated (*N*)*	N**	%^†^	PY	Incidence rate per 100 PY (95% CI)
ALT or SGPT (U/L)										
>3× ULN	536	30	5.6	435	6.9 (2.7–14.3)	344	18	5.2	282	6.4 (3.4–9.3)
>5× ULN		11	2.1	454	2.4 (0.4–7.9)		10	2.9	287	3.5 (1.3–5.6)
>10× ULN		1	0.2	461	0.2 (0.0–4.1)		1	0.3	295	0.3 (0–1.9)
>20× ULN		1	0.2	461	0.2 (0.0–4.1)		1	0.3	295	0.3 (0–1.9)
AST or SGOT (U/L)										
>3× ULN	536	14	2.6	453	3.1 (0.7–8.9)	353	13	3.7	296	4.4 (2.0–6.8)
>5× ULN		4	0.7	459	0.9 (0.1–5.3)		6	1.7	300	2.0 (0.4–3.6)
>10× ULN		1	0.2	461	0.2 (0.0–4.1)		2	0.6	300	0.7 (0.1–2.4)
>20× ULN		1	0.2	461	0.2 (0.0–4.1)		1	0.3	301	0.3 (0–1.9)
ALP (U/L)										
>2× ULN	536	19	3.5	458	4.1 (1.9–10.5)	331	13	3.9	296	4.4 (2.0–6.8)
>3× ULN		9	1.7	460	2.0 (0.2–7.1)		7	2.1	300	2.3 (0.6–4.1)
>5× ULN		1	0.2	461	0.2 (0.0–4.1)		3	0.9	301	1.0 (0.2–2.9)
Serum bilirubin, total (mg/dL)										
>1.5× ULN	537	43	8.0	442	9.7 (4.6–18.0)	399	23	5.8	342	6.7 (4.0–9.5)
>2× ULN		16	3.0	460	3.5 (0.8–9.5)		15	3.8	346	4.3 (2.1–6.5)
>5× ULN		4	0.7	461	0.9 (0.1–5.3)		7	1.8	349	2.0 (0.5–3.5)
>10× ULN		1	0.2	461	0.2 (0.0–4.1)		3	0.8	349	0.9 (0.2–2.5)
LFT combinations of Hy's Law^‡^	535	3	0.6	461	0.6 (0.0–4.9)	285	1	0.3	276	0.4 (0–2.0)

*N evaluated: number of eligible patients with at least one follow-up measure of the corresponding LFT. **N with elevation: number of patients with LFT elevation. ^†^ *N* with elevation/*N* evaluated. ^‡^Hy's Law = ALT or AST > 3× ULN, ALP < 2× ULN, and bilirubin > 2× ULN. TKI: tyrosine kinase inhibitor; ULN: upper limit of normal; ALP: alkaline phosphatase; ALT: alanine aminotransferase; PY: person-years; SGPT: serum glutamic pyruvic transaminase; SGOT: serum glutamate-oxaloacetate transaminase.
